# Monocular Localization with Vector HD Map (MLVHM): A Low-Cost Method for Commercial IVs

**DOI:** 10.3390/s20071870

**Published:** 2020-03-27

**Authors:** Zhongyang Xiao, Diange Yang, Tuopu Wen, Kun Jiang, Ruidong Yan

**Affiliations:** State Key Laboratory of Automotive Safety and Energy, School of Vehicle and Mobility, Tsinghua University, Beijing 100084, China

**Keywords:** vehicular localization, HD map, monocular camera, semantic features, OpenDrive

## Abstract

Real-time vehicle localization (i.e., position and orientation estimation in the world coordinate system) with high accuracy is the fundamental function of an intelligent vehicle (IV) system. In the process of commercialization of IVs, many car manufacturers attempt to avoid high-cost sensor systems (e.g., RTK GNSS and LiDAR) in favor of low-cost optical sensors such as cameras. The same cost-saving strategy also gives rise to an increasing number of vehicles equipped with High Definition (HD) maps. Rooted upon these existing technologies, this article presents the concept of Monocular Localization with Vector HD Map (MLVHM), a novel camera-based map-matching method that efficiently aligns semantic-level geometric features in-camera acquired frames against the vector HD map in order to achieve high-precision vehicle absolute localization with minimal cost. The semantic features are delicately chosen for the ease of map vector alignment as well as for the resiliency against occlusion and fluctuation in illumination. The effective data association method in MLVHM serves as the basis for the camera position estimation by minimizing feature re-projection errors, and the frame-to-frame motion fusion is further introduced for reliable localization results. Experiments have shown that MLVHM can achieve high-precision vehicle localization with an RMSE of 24 cm with no cumulative error. In addition, we use low-cost on-board sensors and light-weight HD maps to achieve or even exceed the accuracy of existing map-matching algorithms.

## 1. Introduction

Precise knowledge of the localization and orientation of a vehicle is critical in realizing many map-based IV applications [1,2], such as decision-making, cooperative driving, map updating, etc. The state-of-the-art integrated inertial navigation system based on the global navigation satellite system (GNSS) real-time kinematic (RTK) module, high-quality IMU, and LiDAR-based positioning technology is well-known to deliver high-precision localization [3]; however, due to its high cost, it remains mostly in the research stage and is seldom seen in mass-produced vehicles.

**Figure 1:**
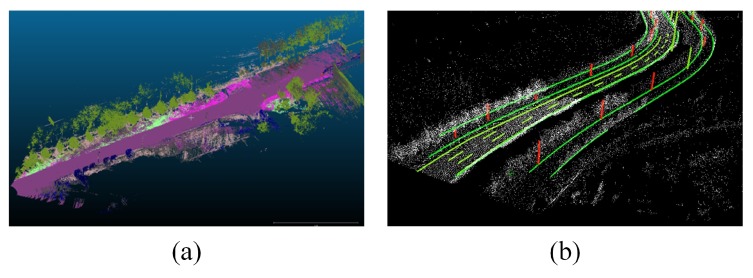
Different types of High Definition (HD) map—(**a**) point cloud map with semantic labels; (**b**) vector HD map of a typical urban road.

As an effective augmentation tool among on-board sensors, the HD compact map has gained tremendous popularity as a consumer vehicle add-on feature. With pre-collected environmental information, the HD map can act as a virtual sensor to improve vehicle safety without incurring additional hardware system complexity [4]. As a result, high-precision HD maps are considered the cornerstone of IV technology, especially for more advanced automated vehicles [2]. Although HD maps are often deployed in costly equipment such as LiDAR and the corresponding high-performance integrated inertial navigation system, the HD map itself is not considered as a costly technology, and thus are widely available as an optional feature for most of the vehicles in the market. Some map-based applications [5,6,7] implement the point cloud maps as shown in Figure 1a. This kind of map retains raw geometric information and may be segmented with semantic labels; however, it is not a market-favorable choice due to its huge data size. On the other hand, Figure 1b shows an example of a vector HD map, of which the refined data are extracted from the raw point cloud map for a smaller data size. It contains the precise geometric descriptions of road elements and retains higher-level semantic information such as road topology, lane type, speed limit, etc. In recent years, with the release of various high-precision map standards such as OpenDrive and NDS, vector HD maps from mainstream cartographers (e.g., TomTom and HERE) have thrived in commercial vehicles.

High-precision absolute vehicle localization is particularly important for HD map-based applications in IVs. For example, the vehicle must be able to localize itself before the information in the map becomes relevant for IV route planning; in some applications such as the Road Experience Management of Mobileye [8], vehicle localization is a prerequisite for pending map updates. GNSS is one of the most commonplace localization technology deployed on vehicles. The RTK technology can even achieve centimeter-level localization by modifying the mobile station with reference station data [9]. Nevertheless, its high accuracy can only be maintained in open areas. On typical urban roads, the accuracy of GNSS RTK struggles to remain stable due to satellite signals suffering from multipath fading and shadow effects by the nearby buildings and vegetation [10,11]. Such GNSS signal degradation issues are dealt with by dead reckoning (DR). With the information from inertial sensors such as gyroscope, accelerometers, or LiDAR odometry, the current position of the vehicle can be effectively inferred [12]. However, the cumulative positional errors can quickly spiral out of control due to the long-term absence of global localization information, especially when compromised by inertial sensors with sub-par performance [13]. This shortcoming of DR is addressed by the map-matching positioning process [14], in which LiDAR is primarily used to collect maps beforehand to generate the raw point cloud maps (as is shown in Figure 1a) with dense features [14,15]; then the point cloud of the vehicular LiDAR is registered by the ICP [16] or NDT [17] method to calculate the accurate vehicle position and rotation with centimeter-level precision. While both families of localization methods—RTK GNSS and DR with LiDAR—offer accurate vehicle localization, both require high-cost in-vehicle sensors. Consequently, their intimidating price point prevents them from becoming truly viable solutions for the IV market. In order to break the price barrier and deliver a market-friendly vehicle localization solution, a completely different design philosophy must be taken. Rather than relying on the high-end hardware, this article proposes to develop a new vehicle localization method based on low-cost visual sensors and vector HD maps, both of which are commonly equipped solutions on Tesla, MobilEye, BOSCH, and Baidu Apollo Lite as cost-down strategies.

The association between the pre-collected map and visual sensors is a key issue in localizing the visual sensor in the map coordinates [18]. There have been many research papers on visual feature points in the SLAM domain. Among them, there are many works based on local features, and many of them have made great basic contributions to robot positioning and mapping, such as Scale-Invariant Feature Transform (SIFT) [19,20], Speeded Up Robust Features (SURF) [21] and ORB [22,23]. These methods can provide stable features under changes of illumination, viewing angle, and scale. In some articles, visual feature points are extracted and matched against point cloud maps [5,24]. However, problems starting to rise due to limited storage and computational power [25]. On the other hand, some scholars have put forward easy-computation global appearance descriptors, which are mainly used for robot positioning and topology map construction [26,27]. Principal Components Analysis (PCA) [28] and Discrete Fourier Transform (DFT) [29] are used for generating global appearance descriptor to concentrate the information of an image in a lower number of components. This kind of description method describes the whole image as a single descriptor, which has advantages in some unstructured or dynamic environments where road landmarks are not easy to be extracted.

In recent years, with the development of deep learning and the improvement of on-board computing ability, the information contained in an image can be interpreted down to pixel-level resolution [30], thereby enabling more complex image feature recognition such as semantic information extraction [31,32]. Compared with traditional visual descriptors, semantic information is more robust against seasonal changes, ambient illumination fluctuations, and occlusions due to dynamic obstacles. In addition, semantic features are more easy to be matched maps storing semantic features. Consequently, it gained some popularity as cues in map matching process [6]. However, all the published semantic matching methods are centered around a stereo vision system composed of either a high-cost specialized camera [33], LiDAR [34], or based on the less-favored point cloud map instead of the vector map. In addition, since deep learning inherently consumes lots of computing resources, the extraction of semantic features in these algorithms takes a lot of time, resulting in limited real-time performance [33,35]. In this paper, one consideration of the integration of visual odometry is that the frame rate is higher, which can compensate the time lag from semantic segmentation in the map localization module, and finally output the localization results in real-time.

In summary, to the best of our knowledge, the alignment between monocular vision and the standardized vector HD map using semantic cues is a promising map-matching vehicle localization solution with lingering commercialization challenges such as weather-dependent reliability, unfavorable cost, and large map data size. Therefore, in order to address these shortcomings, we propose in this paper the concept of Monocular Localization with Vector HD Map (MLVHM), a novel map-based localization algorithm, as well as its data association method implemented on low-cost visual sensors and compact HD vector maps to deliver high-precision, drift-free vehicle localization. The preliminary version of this work was presented in [35]. Compared with the preliminary version, we improved the method of data association between the camera and HD map data. Furthermore, we simulated and verified the accuracy of localization based on single-frame, and analyzed the failure scenarios of the preliminary version as well. We also included frame-to-frame constraints to further improve scene adaptability, positioning accuracy, and real-time performance.

The main contribution of this paper can be elaborated by the following three points:

1. We propose a low-cost and high-precision localization method based on the extraction of semantic vector features and robust map matching algorithm.

2. We propose and demonstrated a sliding-window based frame-to-frame motion fusion to effectively improve the stability of localization in scenes with sparse localization features and enable the real-time performance as well.

3. Finally, we simulate and conduct real-world experiments to fully analyse the accuracy and reliability of the proposed localization algorithm.

The remainder of the paper is organized as follows. The related works are introduced in Section 2. In Section 3, we give an overview of the proposed localization method, and detailed introduction of the basic theoretical methods and application details in this paper are given in Section 4 and Section 5, respectively. In Section 6, we analyze the factors that affect the accuracy of the map-based localization. In Section 7, evaluation and discussion based on real-vehicle test are given. Finally, we conclude the paper with future research efforts in Section 8.

## 2. Related Works

A typical map-based visual localization system usually includes the following components: (1) road maps that are pre-collected offline, (2) Visual features extracted from pre-loaded images for map association, and (3) camera pose estimation algorithm for calculating the camera pose based on the alignment of images and maps. In some studies, frame-to-frame motions are also introduced from inertial sensors to aid the localization. In this section, we will discuss the prior works of each component with a dedicated focus on the localization methods using visual sensors and how these prerequisite works helped with the development of MLVHM.

### 2.1. Maps and Visual Features for Association

Table 1 presents a selected list of representative high-precision localization research works, each with its specific type of required visual features for map matching. Note that only the last two methods use semantic features. In [24], a local map of ORB feature points with SLAM is first generated, followed by associating the local map with a 3D LiDAR point map in order to estimate the camera pose. In [5], SIFT key point features are stored in a 3D point map and aligned with the same feature points extracted from the on-board camera. These two methods use visual feature points which are sensitive to the change of ambient illumination and camera view angle. Consequently, the map-matching process becomes painstakingly difficult when subjected to drastic seasonal weather changes or a significant number of dynamic obstacles in the camera view.

In recent years, however, the development of deep learning algorithms (e.g., lane recognition and pixel-level semantic segmentation) makes it possible to acquire semantics-level features from camera images. Compared with visual feature points, higher-level semantic information is much more resilient against changes in light and camera perspectives. Even if occluded, part of the reference landmarks can be still be effectively identified for subsequent map-matching. Therefore, the concept of semantic feature-based map-matching and vehicle localization has attracted a significant amount of research attention. For instance, in [33], pole-like landmarks are extracted from a stereo camera to associate with a semantic vector map. Ref. [34] matches the map with landmarks extracted from the fused images from both camera and LiDAR systems. Nonetheless, both of these approaches have been implemented not only on cameras but also on other on-board sensors to obtain the in-depth information for relevant features, thus inadvertently require more complex hardware system than our proposed monocular vision algorithm does. In another recent monocular vision localization work [6], labeled 3D point clouds are associated with segmented pixels. However, this approach is not able to accurately describe the geometric features of the landmarks, resulting in relative low localization accuracy. In addition, some of the works mentioned above are based on 3D point cloud maps, most of which are collected directly by LiDAR with abundant geometric information but no semantic level details. Compared to the existing vehicle maps in the market, such as NDS and OpenDrive, vectorized maps adopt vector map format to describe landmarks with control points and semantic labels instead of point cloud maps, and thus are much easier to manage and update.

### 2.2. Data Association and Vehicle Pose Estimation

Data association methods address the information association challenges between map landmarks and features recognized by on-board sensors. For the methods using visual feature points, the binary descriptor of feature points are often used as a clue of association, resulting in a relatively simple association process. On the other hand, for the methods implementing semantic features, some develop the correlation based on the diameter of the landmark [33]. However, in real driving scenarios, many landmarks can appear in repetitive patterns (e.g., light poles), leading to ambiguity in matching. ICP is a widely-used registration method. In some studies [24,36], the map is projected into the camera coordinates based on the camera position, and the matching relationship is estimated iteratively by updating the camera position, assuming that the nearest features are matching pairs. The method nevertheless inevitably introduces the influence of outliers, thus is almost always paired up with the RANSAC method in order to eliminate mismatching and improve accuracy [5,6].

Based on the association of features between maps and the camera, the vehicle pose can be calculated in the map coordinates. Some preliminary methods simplifies the camera motions to the two-dimensional coordinate system models [34,36], making it unsuitable for scenarios where the external parameters (e.g., elevation, height) of the camera change. A much more accurate camera pose description is the six-DOF model, which is adopted in our work and others [6].

As for the vehicle localization, two main methods are used in localization parameter estimations: filtering and optimization. Some common filtering methods are the Kalman Filter [3], the Extended Kalman Filter [36] and the particle filter [33,37]— Monte Carlo localization algorithm—which models the probability of sensor readings in order to predict and update the camera pose. In the works pertaining to optimization method [24], the pose estimation problem is viewed as a maximum likelihood (ML) formula where the sensor readings are compared with the map re-projection results to produce constraints, against which the camera pose is estimated iteratively. Generally speaking, the filtering method has less computational complexity, and the optimization method is more suitable for dealing with non-linear models [38].

### 2.3. Integration of Frame-to-Frame Constraints

Map constraints in many localization methods are rather incomplete. As an example, in [36,39], lane lines can only provide lateral constraints, which is of very limited usability for IVs. In addition, the accuracy of the vehicle localization based solely on map matching methods are prone to sparse map features. To address these shortcomings, the localization framework must incorporate other supplementary information. Besides fusing the absolute localization information such as GNSS, it can also exploit the inter-frame motion information for smoothing. For example, IMU, vehicle dynamic constraints, and wheel odometry have all been integrated in the localization system as map-matching supplements [3,39]. These additional inter-frame constraints can be easily incorporated into the prediction step in the filtering framework. In the optimization framework, residual terms can also be added to the optimization problem to describe these constraints [40].

In this paper, we use the monocular vision odometer as the inter-frame constraint to further improve the localization results of the map-matching method while avoiding the use of additional sensors. Prior to proceeding forward, the difference between the localization problem and the SLAM problem must be clarified. In the domain of SLAM in robotics, the position of the robot is estimated simultaneously with environmental modeling [41]. SLAM is able to estimate the relative pose of the robot with respect to that in the previous frame. Although, it is unable to pinpoint the absolute location of the vehicle on the map. Thus, one key goal in this paper is to deliver a solution for absolute localization of the vehicle in an HD map, i.e., the earth coordinate system. Frame-to-frame motion estimation by SLAM is also integrated, but only as an aid to reduce errors in map-matching.

The problem of re-localization is studied in some SLAM works by setting off a robot to revisit a location around which the environment has been previously modeled. This paper also has a very similar scope of work. In existing works, the mapping sensor and the relocated sensor are required to use the same data type for the simplicity in feature association. For example, in ORB-SLAM, the map formed by the algorithm is a map composed of ORB feature points. When relocating, the ORB feature points in the image of the on-board camera are matched with the historical feature points. However, these feature points are very difficult to be correctly matched at times due to changes of illumination or view angles. On the contrary, this paper focuses on using the higher-level semantic features instead, which are relatively stable under environment changes in ambient illumination, angle of view, season, and weather. More importantly, this approach allows the re-localization mapping to be carried out with different sensors. In the experimental example in this paper, LiDAR is used for mapping; nevertheless, other visual sensors such as depth cameras, binocular cameras, etc. can also be used to produce this map.

## 3. System Overview

**Figure 2:**
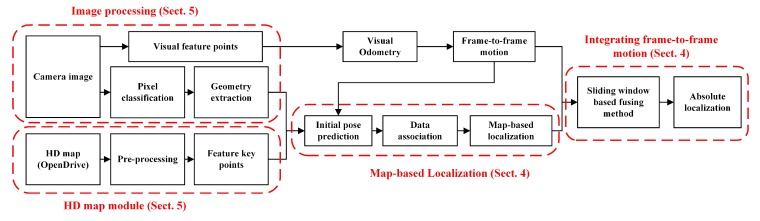
Overview of the proposed method—Monocular Localization with Vector HD map (MLVHM).

Figure 2 illustrates the system overview of the MLVHM. The HD map module is responsible for map data pre-processing and feature control point extraction for the MLVHM localization model from an HD map in OpenDrive format. In order to achieve high accuracy during map data acquisition, LiDAR is deployed in the real vehicle experiments solely for map acquisition; nowhere throughout the experiment was LiDAR used for vehicle localization. Moreover, the only limitation imposed on the experiment setup is the map data format. As long as the acquired maps conform to the requirement, the maps collected by other sensors are also compatible with our localization method.

The image processing module takes care of the geometric feature extraction with the semantic class for image-to-map alignment. First, the pixels are classified semantically through deep learning, followed by clustering the key pixels critical to the map matching process. Then, these key pixel clusters are point- or line-fitted to help extract the geometric features with semantics. At the same time, based on the SLAM algorithm, the ORB features are extracted for estimating the relative motion of the camera. It is noteworthy that the ORB features extracted here do not participate in map matching, but are only used for motion tracking.

In the map-based localization module, the camera pose is estimated based on map-visual semantic feature matching. The data association sub-module contains the algorithm for determining the optimal matching based on the consistency of random sampling, while making adaptive improvements according to the driving scenes. After the landmarks have been associated with the image features, an objective function is defined to describe the constraints of map-visual semantic features, and the camera pose is solved iteratively through the optimization algorithm.

In the analysis, it is found that the localization is unstable in some sparse scenes, and the map localization module can not guarantee the real-time localization output since the calculation for semantic segmentation takes too much time. In order to further improve the stability and accuracy as well as to guarantee the real-time output of localization results, a sliding window-based method is deployed to fuse the original global pose with the frame-to-frame motion before outputting the final result—an absolute vehicle pose of 6-DOF in the map coordinates.

## 4. Map-Based Localization

This section covers the design and analysis of the proposed camera pose estimation method based on the matched geometric features with semantics. This analysis is fully generalized and not biased towards certain objects or features in a given traffic scene; however, a concrete implementation example will be provided in Section 5. This section further covers the novel data association method between the camera image and the map features, as well as the integration of inter-frame constraints to further improve the localization stability.

### 4.1. Line- and Point-Based Camera Localization

**Figure 3:**
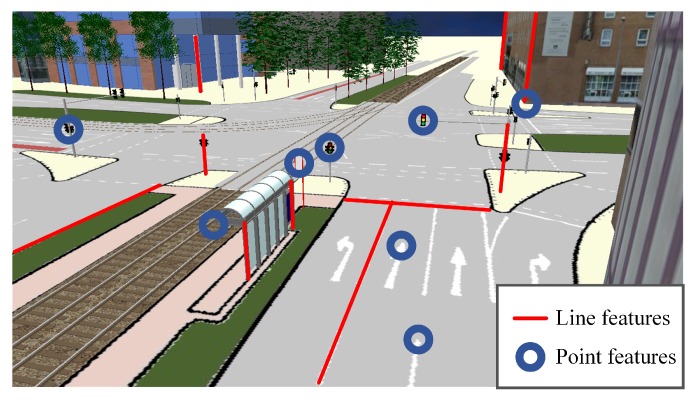
Illustration of road features. Red lines represent line features for localization reference; blue circles represent point features.

Throughout the analysis, a feature refers to an object captured by an on-board camera, and a landmark is an object extracted from a pre-collected map. The features adopted by the MLVHM are geometric features with semantics, as shown in Figure 3. In traffic scenes, the extracted geometric features include but not limited to line features, light poles, lane lines, edge of walls, edges of selected road signs, etc. Selected point features are also extracted, including, but are not limited to the center of traffic signs, corner points of buildings, markers on the ground, etc. In image processing, these point and line features are identified with semantic information (e.g., light poles identified as light pole lines, lane as lane lines, etc.). Correspondingly, the landmarks stored in the map are also geometric descriptions of the key elements of the environment.

The problem of solving for the camera localization can be described as a state estimation problem: Given the observation of features $Z_{t}$, landmarks M and the correspondences c, the camera pose at time *t* $x_{t}$ is estimated.

The six-DOF model is deployed to describe the position and orientation of the camera in the map coordinate system, that is, the world coordinate system:

(1) $$\begin{array}{c} x_{t}=\underset{C_{t}}{\underset{︸}{[x_{t},y_{t},z_{t}}},\underset{A_{t}}{\underset{︸}{\theta_{t},\psi_{t},\phi_{t},]}} \end{array}$$

where $C_{t}$ and $A_{t}$ are the localization and orientation parameters, $x_{t}$, $y_{t}$, and $z_{t}$ are the three coordinates of the optical center of the camera, and $\theta_{t}$, $\psi_{t}$, and $\phi_{t}$ are respectively the pitch, yaw, and roll angle of the camera in the map coordinates.

Given a set of line and point features extracted from the on-board camera as the observation at time *t*:

(2) $$Z_{t}={\left\{z_{i}^{\left(P\right)},z_{m}^{\left(L\right)}\right\}}_{i=1:N_{FP},m=1:N_{FL}}$$

where $N_{FP}$ and $N_{FL}$ are the number of point and line features, $z_{i}^{\left(P\right)}\in R^{2\times 1}$ is the coordinate of point feature in the pixel coordinate system, and $z_{m}^{\left(L\right)}\in R^{4\times 1}$ is composed of the coordinates of the two endpoints of a given line feature.

The landmarks from the HD map is defined as,

(3) $$M={\left\{m_{j}^{\left(P\right)},m_{n}^{\left(L\right)}\right\}}_{j=1:N_{LP},n=1:N_{LL}}$$

where $N_{LP}$ and $N_{LL}$ are the number of point and line landmarks, respectively, and $m_{j}^{\left(P\right)}\in R^{3\times 1}$ is the 3D coordinate of the point landmark. The two end-point $P_{n,1}^{\left(P\right)}$ and $P_{n,2}^{\left(P\right)}$ are used to describe the line landmark. Therefore,

(4) $$m_{n}^{\left(L\right)}=\left[\begin{array}{c} P_{n,1}^{\left(L\right)}\\ P_{n,2}^{\left(L\right)} \end{array}\right]$$

and $m_{n}^{\left(L\right)}\in R^{6\times 1}$.

Lastly, the correspondence is defined as $c=\left\{c^{\left(P\right)},c^{\left(L\right)}\right\}$, where $c^{\left(P\right)}=\left\{i,j\right\}$, indicating that the *i*th point feature extracted from the image is associated with the *j*th point landmark in the HD map. Similarly, $c^{\left(L\right)}=\left\{m,n\right\}$ can be subsequently defined.

The measurements model of line features is:

(5) $$z_{m}^{\left(L\right)}=h^{\left(Line\right)}\left(m_{n}^{\left(L\right)},x_{t}\right)+v_{t}^{\left(L\right)},$$

where $\left(m,n\right)\in c^{\left(L\right)}$. $h^{\left(Line\right)}\left(m_{n}^{\left(L\right)},x_{t}\right)\in R^{2\times 1}$ is the measurement of the *n*th line landmark at camera pose $x_{t}$. The measured noise is assumed to obey the Gaussian distribution, i.e., $v_{t}^{\left(L\right)}\in N\left(0,R_{t}^{\left(L\right)}\right)$, which describes the uncertainty of extracting the line features.

Similarly, the point measurements model is:

(6) $$z_{i}^{\left(P\right)}=h^{\left(Point\right)}\left(m_{j}^{\left(P\right)},x_{t}\right)+v_{t}^{\left(P\right)},$$

where $\left(i,j\right)\in c^{\left(P\right)}$. $h^{\left(Point\right)}\left(m_{j}^{\left(P\right)},x_{t}\right)\in R^{2\times 1}$ is the measurement of the *j*th point landmark at camera pose $x_{t}$. $v_{t}^{\left(P\right)}\in N\left(0,R_{t}^{\left(P\right)}\right)$, which altogether describes the uncertainty of point feature extractions in the image processing.

The pin-hole camera model $\pi :\left(R^{3},R^{6}\right)\to R^{2}$ is applied to project the control points p to the image coordinate $u={\left[u,v\right]}^{T}$ based on the camera pose: $x_{t}$.

(7) $$\pi :\left[\begin{array}{c} u\\ 1 \end{array}\right]=KR\left(A_{t}\right)\left[I|-C_{t}\right]\left[\begin{array}{c} p\\ 1 \end{array}\right]$$

where K is the intrinsic matrix of the camera, $R(A_{t})$ is the rotation matrix from the world coordinate system to the camera based on the camera orientation. Subsequently, in the Line measurement $h^{\left(Line\right)}$, the measurement model is expressed as:

(8) $$h^{\left(Line\right)}\left(m_{n}^{\left(L\right)},x_{t}\right)=\left[\begin{array}{c} \pi (p_{n,1}^{\left(L\right)},x_{t})\\ \pi (p_{n,2}^{\left(L\right)},x_{t}) \end{array}\right]$$

and the point measurement $h^{\left(Point\right)}$ is expressed as:

(9) $$h^{\left(Point\right)}\left(m_{j}^{\left(P\right)},x_{t}\right)=\pi \left(p_{j}^{\left(P\right)},x_{t}\right)$$

Therefore, the task of localizing the camera in the map is ultimately estimating $x_{t}$ from the measurements $Z_{t}$, which are assumed to be probabilistically independent conditioned on $x_{t}$.

The camera state thereby can be estimated by applying the maximum likelihood method:

(10) $$\begin{array}{cc} {\hat{x}}_{t} & =\underset{x_{t}}{\arg\max}P\left(Z_{t}|x_{t}\right)\\ \; & =\underset{x_{t}}{\arg\max}\left(\prod_{i}P\left(z_{i,t}^{\left(P\right)}|x_{t}\right)\prod_{m}P\left(z_{m,t}^{\left(L\right)}|x_{t}\right)\right) \end{array}$$

As the observation noise is assumed to conform to the normal distribution, based on the measurement models (5) and (6),

(11) $$\begin{array}{cc} \; & P\left(z_{i,t}^{\left(P\right)}|x_{t}\right)=N\left(h^{\left(Point\right)}\left(m_{i}^{\left(P\right)},x_{t}\right),R_{t}^{\left(P\right)}\right) \end{array}$$

(12) $$\begin{array}{cc} \; & P\left(z_{m,t}^{\left(L\right)}|x_{t}\right)=N\left(h^{\left(Line\right)}\left(m_{m}^{\left(L\right)},x_{t}\right),R_{t}^{\left(L\right)}\right) \end{array}$$

where $R_{t}^{\left(P\right)}$ and $mathbfR_{t}^{\left(L\right)}$ are the covariance matrices of the feature recognition noise. Considering (11) and (12), the maximum likelihood estimation problem (10) can be transformed into the following non-linear optimization of Mahalanobis norm of all measurement residuals:

(13) $$\begin{array}{c} r\left(x_{t}\right)=\sum_{i}{∥\begin{array}{c} r^{𝒫}\left(z_{i,t}^{\left(P\right)},x_{t}\right) \end{array}∥}_{R_{t}^{\left(P\right)}}+\sum_{m}{∥\begin{array}{c} r^{\left(ℒ\right)}\left(z_{m,t}^{\left(L\right)},x_{t}\right) \end{array}∥}_{R_{t}^{\left(L\right)}} \end{array}$$

(14) $${\hat{x}}_{t}=\underset{x}{\arg\min}r\left(x_{t}\right),$$

where $r^{𝒫}\left(z_{i,t}^{\left(P\right)},x_{t}\right)$ and $r^{\left(ℒ\right)}\left(z_{m,t}^{\left(L\right)},x_{t}\right)$ are residuals of point and line measurements respectively.

**Figure 4:**
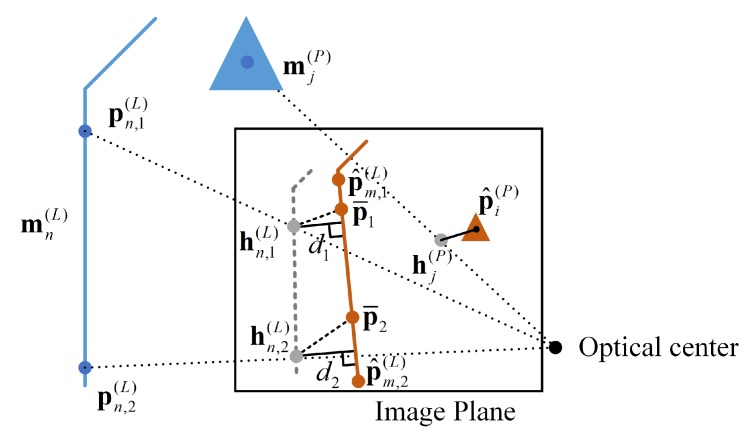
Definition of residuals. The landmarks in space denoted in blue; the recognized feature in gold; and the re-projection of map features in grey.

As is shown in Figure 4, suppose the re-projected point of point landmark $m_{j}^{\left(P\right)}$ is $h_{j}^{\left(P\right)}$, and the corresponding control point extracted from the image is point ${\hat{P}}_{i}^{\left(P\right)}$ ($\left(i,j\right)\in c^{\left(P\right)}$), the residual of point features can then be expressed as

(15) $$r^{𝒫}\left(z_{i,t}^{\left(P\right)},x_{t}\right)=h_{j}^{\left(P\right)}-{\hat{P}}_{i}^{\left(P\right)}.$$

The line feature residuals are approximated due to the ambiguity in identifying the points in the image that corresponds to the two control points in the map. As is shown in Figure 4, given a line landmark $m_{n}^{\left(L\right)}$, and its two control points in the map as $P_{n,1}^{\left(L\right)}$ and $P_{n,2}^{\left(L\right)}$, their corresponding re-projected points on the image plane of camera pose $x_{t}$ are $h_{n,1}^{\left(L\right)}$ and $h_{n,2}^{\left(L\right)}$ as a result. If the line feature is to be extracted and associated with this landmark ($\left(m,n\right)\in c^{\left(L\right)}$) during image processing, the two resultant control points of the extracted feature would then be ${\hat{P}}_{m,1}^{\left(L\right)}$ and ${\hat{P}}_{m,2}^{\left(L\right)}$. However, the true points on the extracted line is ${\bar{P}}_{1}$ and ${\bar{P}}_{2}$. The residuals of points $\bar{h_{n,1}^{\left(L\right)}{\bar{P}}_{1}}$ and $\bar{h_{n,2}^{\left(L\right)}{\bar{P}}_{2}}$ are approximated by $d_{k},\left(k\in \left\{1,2\right\}\right)$, the distance from two re-projected map control points to the recognized line, where,

(16) $$d_{k}=\frac{|a_{m}u\left(h_{n,k}^{\left(L\right)}\right)+b_{m}v\left(h_{n,k}^{\left(L\right)}\right)+c_{m}|}{\sqrt{a_{m}^{2}+b_{m}^{2}}}$$

where $\left(a_{m},b_{m},c_{m}\right)$ are the parameters of the line segment $\bar{{\hat{p}}_{m,1}^{\left(L\right)}{\hat{p}}_{m,2}^{\left(L\right)}}$, and the residual of line features is,

(17) $$r^{\left(ℒ\right)}\left(z_{m,t}^{\left(L\right)},x_{t}\right)=\left[\begin{array}{c} d_{1}\\ d_{2} \end{array}\right]$$

### 4.2. Data Association Method

The data association module solves the problem of correspondence between map landmarks and detected features in the observation model. Although the semantic information of the features recognized in vision is a very helpful association clue, repetitive visual semantic features, such as recurring road signs, still present tremendous difficulties in the existing semantic-based matching processes.

The novel data association method in this paper is an improved version of the RANSAC method. The basic idea is to randomly extract a subset of possible matching sets with the same semantics, and evaluate the quality of this subset. In basic RANSAC, the quality of subsets is measured by the number of inliers that match the model; that is, the number of matches within a certain threshold that can be obtained by projecting the position and attitude of the map to the pixel coordinate system. In the improved RANSAC method presented in this paper, the drift of the optimized position and pose away from the initial position which helps improve the robustness of the matching algorithms is deployed in conjunction with the basic RANSAC. To illustrate the association method, the algorithm pseudocode is provided in Algorithm 1.

In order to validate the algorithm, a possible correspondence set is randomly generated, and the line features with the same semantics are randomly matched within the set. Three line correspondences (which is the minimal set of correspondences to calculate the camera pose) are randomly sampled and plugged into the Equation (14) for determining the camera position and pose, from which the landmarks in the map can be projected into the camera. If the distance between the landmark and the feature identified in the camera is less than the set threshold, it is considered as an inlier. In addition, the drift from the estimated pose to the initial guess is calculated. If the respective drift satisfies the conditions outlined in step 6 in Algorithm 1, the hypothesized correlation is added to the association hypothesis C. *D* is the distance threshold of the localization drift between the localization result and initial pose prediction, which is set according to the confidence of the initial guess of the camera pose. The initial pose the first frame is from the low-cost GNSS, and the subsequent ones are predicted based on the positioning result of the previous frame and the Visual Odometry. In our test, *D* for the first frame is set to 6 m, while those for the subsequent frames are set to 1 m. The line 10 in Algorithm 1 shows how to find the best association from a bunch of association groups. Our approach is to calculate the number of association pairs in each group, that is, the size of the inliers, and find the one with the largest number of inliers as the final association.
**Algorithm 1** Data association**Input:** Initial camera position $\bar{p}$; HD Map M; Extracted features F;**Output:** Correspondence c1: $c_{0}=Possible\_correspond\left(M,F\right)$2: **for** each $c_{1∼3}^{\left(L\right)}\in c_{0}$
**do**3:  $\hat{c}=c_{1∼3}^{\left(L\right)}$4:  Calculate ${\hat{x}}^{*}$ based on $\hat{c}$ according to Equation (14).5:  $c^{*}=Closest\_correspond\;(M,F,{\hat{x}}^{*})$6:  **if**
${∥\begin{array}{c} {\hat{x}}^{*}-\bar{p} \end{array}∥}_{1}<D$
**then**7:   $C=C\cup c^{*}$8:  **end if**9: **end for**10: c=C(size(C)==MAX(size(C)))

### 4.3. Integrating Frame-to-Frame Motion

**Figure 5:**
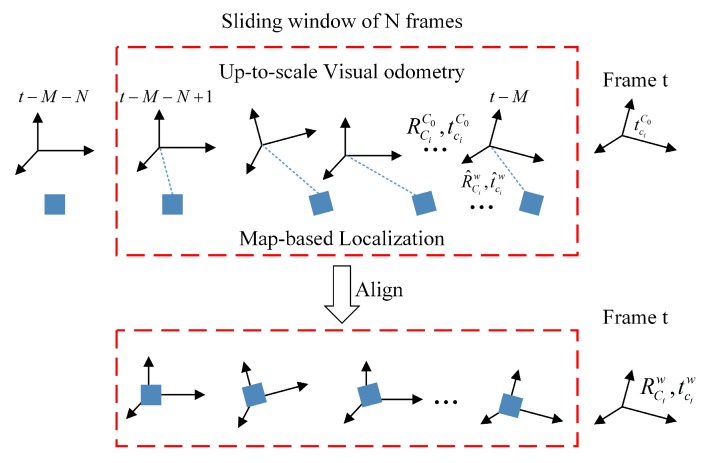
The frame-to-frame motion and map-based localization alignment.

The other branch in the MLVHM system diagram is designed to estimate the odometer readings of the camera using the ORB-SLAM algorithm [23]. This estimation is integrated with the map-based localization results in order to improve the localization accuracy. As shown in Figure 5, the translation vector and rotation matrix of each frame $c_{i}$ is estimated relative to the first frame $C_{0}$ as $t_{c_{i}}^{C_{0}}$ and $R_{c_{i}}^{C_{0}}$. Note that due to the uncertainty of the monocular depth, the translation matrix is up-to-scale - that is to say, the scale of $t_{c_{i}}^{C_{0}}$ differs from the true translation by a factor *s* of one position. As the frame-to-frame motion coordinate system is different from that of the map-based localization result, the two coordinate systems must first be aligned via ORB-SLAM before the processed results can be projected on to the earth coordinate system. With a given rotation and translation matrix $R_{C_{0}}^{w}$ and $t_{C_{0}}^{w}$, the translation vector of a frame $c_{i}$ relative to its subsequent frame $C_{0}$ can be transformed to the earth frame:

(18) $$t_{c_{i}}^{w}=R_{C_{0}}^{w}t_{c_{i}}^{C_{0}}+t_{C_{0}}^{w}.$$

The rotation of frame $c_{i}$ relative to its subsequent one $C_{0}$ is:

(19) $$R_{c_{i}}^{w}=R_{C_{0}}^{w}R_{c_{i}}^{C_{0}}.$$

The frame-to-frame alignment can be achieved by determining $R_{C_{0}}^{w}$ and $t_{C_{0}}^{w}$. Considering the time-consuming nature of image semantic segmentation, the results of map localization generally differ from the current frame time, thus we assume that there is a delay of *M* frames. Firstly, a sliding window of *N* frames is built from t−M−N+1 to t−M, where t−M is the latest frame from map-based localization. For the localization framework formed by our semantic segmentation algorithm, M is usually 6–8. Then, the constraints of each frame in the frame-to-frame motion and map-based localization is calculated within the sliding window, from which the optimization problem can be solved:

(20) $$\begin{array}{cc} \; & {\hat{R}}_{C_{0}}^{w},\hat{t_{C_{0}}^{w}},\hat{s}=\underset{R_{C_{0}}^{w},t_{C_{0}}^{w},s}{\arg\min}\sum_{i=t-M-N+1}^{t-M}\\ \; & \left({∥\begin{array}{c} R_{C_{0}}^{w}t_{c_{i}}^{C_{0}}*s+t_{C_{0}}^{w}-\hat{t_{c_{i}}^{w}} \end{array}∥}_{2}+{∥\begin{array}{c} R_{C_{0}}^{w}R_{c_{i}}^{C_{0}}*{\hat{R_{c_{i}}^{w}}}^{T}-I \end{array}∥}_{2}\right) \end{array}$$

where the first term is the translation constraint (18), and the second is the rotation (19). $\hat{t_{c_{i}}^{w}}$ and $\hat{R_{c_{i}}^{w}}$ are respectively the translation vector and rotation matrix calculated based on the position and attitude angle estimation ${\hat{x}}_{t}$ derived from the map-based localization. Consequently, the rotation and translation matrix of frame t are

(21) $$R_{C_{t}}^{w}={\hat{R}}_{C_{0}}^{w}R_{c_{t}}^{C_{0}}$$

(22) $$t_{C_{t}}^{w}={\hat{R}}_{C_{0}}^{w}t_{c_{t}}^{C_{0}}*\hat{s}+\hat{t_{C_{0}}^{w}}$$

In practice, the size of the sliding window increases gradually from the beginning, but is capped at a selected length in order to reduce the computational complexity. This process realizes the fusion of local localization and absolute localization, and can also restore the scale of monocular SLAM.

## 5. Implementation

All traffic scenarios offer an abundance of point and line features that can serve for MLVHM as well as other similar algorithms developed in recent years. In this section, an example implementation of the MLVHM localization method is demonstrated using light poles and lane line as the line feature, and the center of traffic signs as the point feature. More specifically, this example deploys the method of extracting the semantic geometric features from the image, the method of extracting control points from the map, as well as the details of other algorithm applications. While MLVHM is designed to recognize a greater number of features than presented in this example, this paper will not focus on the feature recognition performance of MLVHM.

### 5.1. Semantic Geometry Feature Extraction

For line features, the lamp poles and lane lines are extracted such that the observation of line features can be rewritten as,

(23) $$z_{j}^{\left(L\right)}=z_{j}^{\left(\xi \right)},\xi \in \left(\mathrm{Pole},\mathrm{Lane}\right)$$

and for point features, the center of traffic signs are extracted accordingly,

(24) $$z_{i}^{\left(P\right)}=z_{i}^{\left(\mathrm{Sign}\right)}.$$

In the process of feature recognition, PSPnet [30] is deployed to semantically segment the image, thereby effectively dividing the pixels of pole-like objects and traffic signs. In order to identify lane lines, a more compact segmentation network is implemented based on the common encoder-decoder architecture, which down-samples the target image 16 times by four convolution layers and decodes with two up-sampling modules; the feature map is up-sample four times within the same process. The entropy loss is applied as the loss function [35]. Because the training samples of segmentation network cover the data of road signs in various lighting, seasonal, and occluded conditions, compared with visual feature points, semantic vector features are more insensitive to seasonal changes and occlusion.

After getting the prediction map of each kind of semantics, we use a certain threshold of possibility to extract the pixels that are likely to belong to a certain kind, and then divide them into several blocks by region growing. After the prediction map of all the relevant semantics is acquired, a selected possibility threshold is applied to extract the pixels with high probabilities as the constituents of one of the key semantic features. These feature-critical pixels are then divided into several blocks by region growing. The line features are extracted by first filtering out blocks with an aspect ratio less than a defined threshold (i.e., not appearing line-like), followed by extracting the remaining lines from blocks by least-square fitting. For light poles, the bent portion at the top is neglected for computation simplicity. Sign-like features are fitted into point elements with its geometric center as the control point.

### 5.2. Utilizing the Compact Map

As mentioned previously, all the map data is organized and saved in OpenDrive format. The landmark geometric control points are extracted from the OpenDrive data and applied to the localization process. Roadside objects are represented by their respective bounding boxes.

Sign-like landmarks have their assigned control points $P_{k}^{\left(S\right)}$ at its geometric center, and are described as $m_{k}^{\left(S\right)}=P_{k}^{\left(S\right)}$. A pole-like landmark (e.g., a light pole) often consists of a pole, a lamp holder, and some custom connection parts with irregular geometries. Only the straight pole portion of the pole-like landmarks are modeled with the two control points at the centers of the upper and lower bounding box surfaces, respectively denoted as $P_{i,1}^{\left(P\right)}$ and $P_{i,2}^{\left(P\right)}$. Subsequently, the pole-like landmarks can be expressed as $m_{i}^{\left(P\right)}=\left\{P_{i,1}^{\left(P\right)},P_{i,2}^{\left(P\right)}\right\}$.

OpenDrive supports lane line representation with polylines. To take advantage of this, polylines with control points placed every 0.2m are used to model the lane lines as $m_{i}^{\left(L\right)}=\left\{P_{i,1}^{\left(L\right)},\cdots P_{i,N}^{\left(L\right)}\right\}$, where $N_{i}$ is the number of control points on the lane. These sampled control points are first filtered based on the selected areas in front of the vehicle, followed by line-fitting into a straight line. This way, the lane lines can be imported into the map model as solid line segments rather than a series of broken lines.

### 5.3. Optimization Method and Initial Values

The Levenberg-Marquardt method is deployed in MLVHM for solving the optimization problem (14) and (20). The residuals are minimized towards zero by iterations:

(25) $$X_{k+1}\leftarrow X_{k}-{\left(J^{T}J+\lambda diag\left(J^{T}J\right)\right)}^{-1}J^{T}f\left(X_{k+1}\right),$$

where λ is determined by the Levenberg-Marquardt method; f is the cost function of each minimization problem; J is the Jacobian matrix of f with respect to the variable to be optimized.

For the initial localization, the current frame localization result is taken as the initial value based on the combination of the absolute localization and SLAM of the previous frames. If no historical frames are available upon algorithm startup, the initial pose is obtained from the low-cost GNSS.

## 6. Analysis of the Relationship between Localization Accuracy and Scenarios

The effectiveness of map-based localization part of MLVHM is verified with a virtual recreation of a real-world road environment with typical landmark density. The very same real-world road environment is used in the real vehicle data test, from which the experimental results can be effectively compared with the simulation, thereby validating all the modules of the MLVHM in typical complex road scenarios. Furthermore, the map-based localization part is subjected to simulations with different semantic feature density and distribution patterns, thereby evaluating its limitations in challenging localization scenarios.

### 6.1. Simulation in Typical Scenario

**Figure 6:**
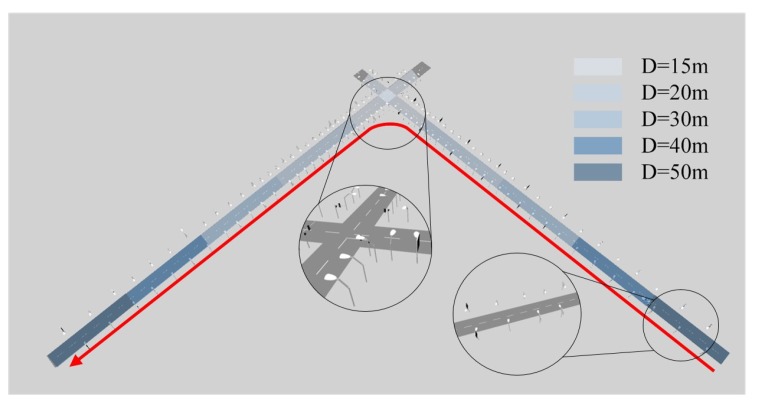
Virtual traffic scenario in simulation setup. The red line denotes the vehicle trajectory.

As shown in Figure 6, a typical urban traffic scenario is deployed with two 800-meter two-way multi-lane roads forming an intersection. The street lamp poles are placed on both sides of the road, with increasingly smaller gaps between poles towards the intersection; the selected gap sizes are 15, 20, 30, 40 and 50 m. In addition, random traffic signs are placed on both sides of the road every 80 m. The car is set to enter from one end of the road, turn left on the intersection, and drive out of the scene. The planned route of the vehicle is shown in the same figure with a red line, along which a total number of 900 frames are collected.

**Figure 7:**
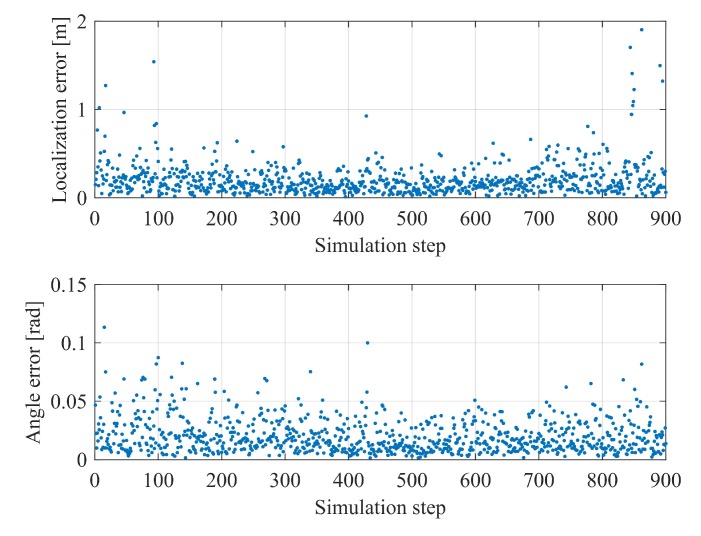
Horizontal localization (top row) and angle errors (bottom row) from simulation result.

Based on the accuracy of common sensors and maps, the maps in the simulation is assumed with a standard deviation of 0.05 m. In the perception results, Gaussian white noise is applied with the line feature offset by the noise of 2 pixels with standard deviation and rotation noise of 0.01 rad, and the point feature similarly by 2 pixels. These noises are independently generated and introduced to the system. In the application of intelligent vehicles, the positioning accuracy in the horizontal plane and the accuracy of direction angle are concerned. As many did in previous studies, we give the horizontal localization error and the angle error are given in Figure 7. The overall localization and attitude angle RMSE are 0.28 m and 0.02 rad, respectively.

**Figure 8:**
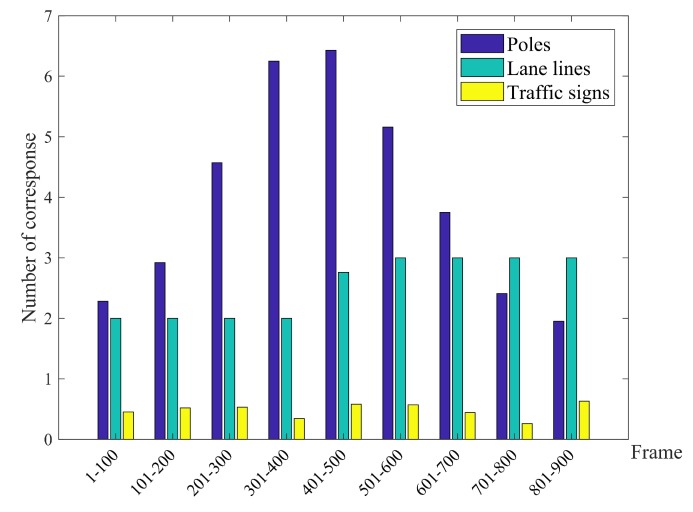
Corresponding road features for every 100 frames.

Figure 8 illustrates the average number of valid semantic features used for map-matching per 100 frames. It is obvious that the density of features will affect the accuracy of positioning results. In Section 6.2, we will do the simulation in more scenes and give further discussion the influence of the distribution of different features on the positioning results.

### 6.2. Localization Performance in More Scenarios and Discussion

**Figure 9:**
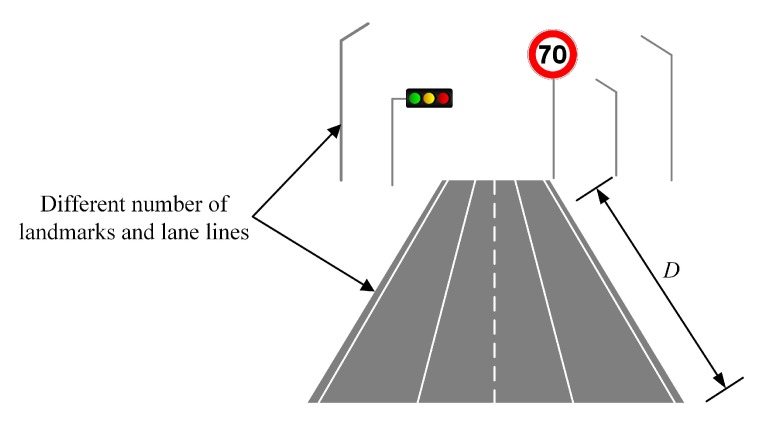
Illustration of scenario definition.

Both simulations and experiments have shown that the localization results vary across different scenarios with different semantic feature distributions. There are endless driving scenarios for vehicles. In order to simulate these scenes more efficiently and determine the performance limitations of the map-based localization part in MLVHM, we quantify the variation of driving scenarios. As shown in Figure 9, three key characteristics are taken into account: horizontal line features (e.g., lane line), vertical line features (e.g., lamp pole), and point features (e.g., road signs). The map-based localization part is run under multiple simulation scenarios with a different number of landmarks and vehicle starting positions; traffic signs and light poles are placed at the end of the road. The distance between the vehicle and these landmarks are denoted as *D*.

**Figure 10:**
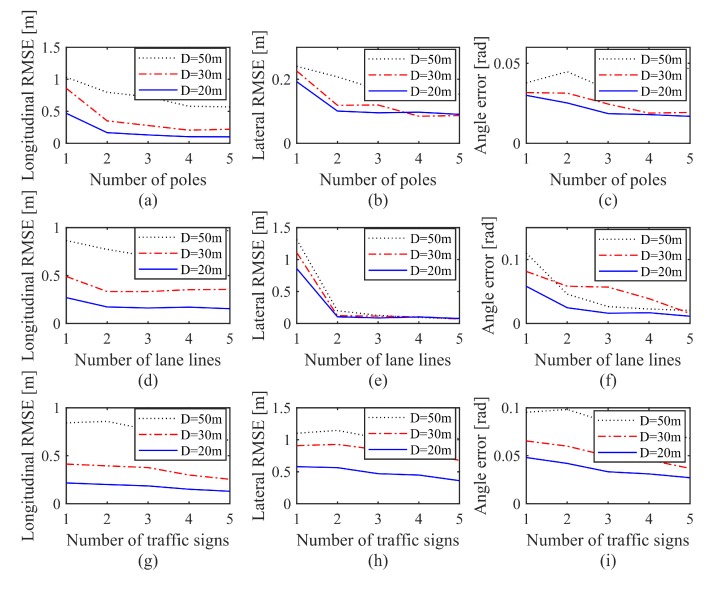
Localization results under different scenarios. (**a**–**c**): effect of vertical line features; (**d**–**f**): effect of horizontal line features; (**g**–**i**): effect of point features.

Similar to the previous simulation setup, the Gaussian noise is introduced to the map and the perception results in this simulation. Three hundred Monte Carlo experiments were carried out for each scene, and all the scene-specific localization RMSEs in different directions of the vehicle were counted. The simulation results of translation and orientation error under different scenarios are shown in Figure 10.

#### 6.2.1. Effect of Vertical Line Features

With the two-lane simulation setup, the vehicle localization accuracy is measured in horizontal and angular errors under different lamp pole counts and their respective distances from the vehicle, as shown in Figure 10a–c.

Overall, the map-based localization part algorithm sees a performance improvement in both translation and orientation with an increased number of lamp poles and shortened *D*. As clearly illustrated in Figure 10a,b, the longitudinal and lateral localization accuracy can respectively reach <50 cm and <20 cm when more than two lamp poles are present in the scene, or *D* is close to 30 m. Some abnormal data points show a slight increase in localization errors when the number of features increases or *D* decreases. This unexpected behavior is well within the error tolerance and only emerged when the localization performance is approaching its maximum, where further increases in feature formation constraints yield only marginal performance improvements.

#### 6.2.2. Effect of Horizontal Line Features

In this simulation, two constant vertical lines are deployed in the setup, from which the impact of horizontal line counts on the localization results can be studied, as shown in Figure 10d–f. By comparing (d) against (e), a highly intuitive conclusion is that horizontal line features parallel to vehicle head direction exhibit a much higher influence on lateral localization than to longitudinal localization. In fact, it is very much conceivable that the constraints formed by the lane line are not sensitive to the movement of the vehicle driving down the lane. On the other hand, changing the lateral position of the vehicle will cause significant changes in the re-projection error. Generally speaking, with fewer features of other kinds, a two-lane road can easily enable vehicles to localize itself down to the decimeter level, but the localization performance improvement is marginal with the increase in the number of lanes. This result is also highly consistent with that from the previous simulation setup, as well as with that from the real vehicle experiment.

#### 6.2.3. Effect of Point Features

For this simulation, three constant light poles are set up as line features, and the impact of the number of traffic signs on the localization results is subsequently studied, as shown in Figure 10g–i. The result indicates clearly that the localization accuracy is much more sensitive to *D* than to the quantity of the traffic signs. In addition, by removing the lanes from the simulation scenario, the result of longitudinal localization is better than that of lateral localization. Compared to the previous simulation data, the line constraints indeed have a higher influence on vehicle localization than the point constraints.

In summary, the localization accuracy is sensitive to feature distributions in a given scenario, and can be improved with an increased number of features as well as a larger distance from the vehicle to the reference landmarks. Through the analysis above, in order to achieve decimeter-level accuracy, four-line features—or three-line features and one additional point feature—are minimally required in the onboard camera vision. Of course, this minimal requirement can be easily fulfilled in general traffic scenarios.

### 6.3. Computational Complexity with Respect to Feature Density

The computational complexity of the algorithm increases as localization feature density increases, since more localization features mean more Jacobian matrices of residual terms to calculate. Assume that *N* features are involved in solving the pose in a single frame, the computational complexity of solving the Jacobian matrix can be approximated as O(N).

**Figure 11:**
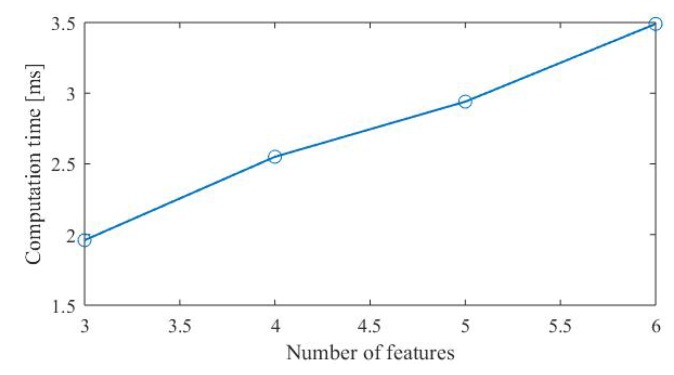
The relationship between the calculation time and the number of features.

We tested the time-consuming optimization calculation on a computer with an Intel (R) Xeon (R) E5-2620 v4 @ 2.10GHz processor, as shown in Figure 11. It can be seen that as the number of features increases, the calculation time increases in a linear fashion, which is consistent with our analysis. In most cases on the real-world scenarios, the number of features is between 4 to 6, so the pose estimation will not cost too much time. The most time-consuming part of the whole process is semantic feature detection. We introduce a visual odometer to ensure real-time localization. As for the real-time performance of the whole algorithm, we will give the experimental results in the experimental part.

## 7. Evaluation

### 7.1. Real Vehicle Experiment in Typical Scenarios

**Figure 12:**
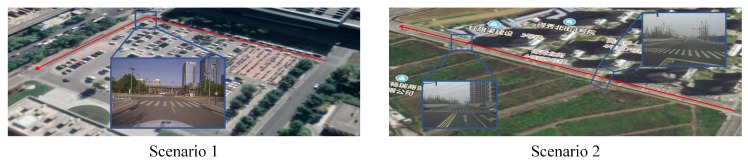
The experimental scenarios.

We conducted real vehicle tests in two typical scenarios with two intelligent vehicles. As shown in Figure 12, the first scenario is a campus scene, in which there are many vehicles parked on the roadside and landmarks such as light poles, traffic signs, etc. are dense. The track of the test vehicle is shown in the red line, which passes through two straight sections and one intersection. The second scene is a typical urban road. Compared with the first scene, there are more lane lines, and the distance between the poles is larger. During the experiment, the vehicle passed three straight roads and two intersections.

**Figure 13:**
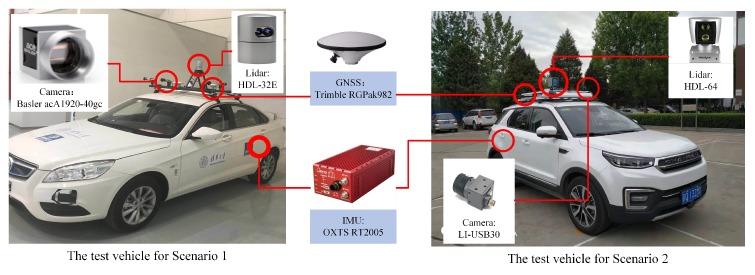
The experimental intelligent vehicles.

As shown in Figure 13, the test vehicle for Scenario 1 is equipped with a 32-line LiDAR and an integrated GNSS system for acquiring the HD map data and reference vehicle trajectory. A Basler industrial camera is used to acquire images for localization. The test vehicle for Scenario 2 is equipped with a 64-line LiDAR, an integrated GNSS system and a USB digital camera for localization validation. We equip each vehicle with a u-blox GPS module (not shown in the figure) with low precision as the initial value for the first frame localization. During each test, the GNSS signal is assumed absent, such that the localization is completed purely by the map and camera vision.

Like other works on vehicle localization, it is difficult to get the true value of the localization, because the positioning data from the integrated GNSS system is not accurate, especially when driving through the buildings and trees shown in the first scene. In this paper, we use the trajectory of LiDAR as the reference trajectory to verify the camera localization results. The external parameters between LiDARs and the on-board cameras are calibrated in advance.

#### 7.1.1. Map Generation

**Figure 14:**
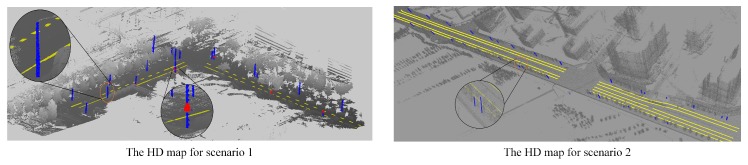
The generated maps used in MLVHM. Lamp poles denoted in blue, lane lines in yellow, and traffic signs in red.

Firstly, the LOAM algorithm is deployed to generate the point cloud map of the road, as shown in Figure 14. Note that the camera data and the map data are collected independently, thus ensuring the localization result of the camera data is independent of map data. The manually extracted lane line, light pole, and traffic signs are denoted in yellow, blue, and red, respectively. Then, their geometric feature points are extracted using the methods from section V, thereby yielding the extracted vector map. The maps are stored in ASCII format with a size of about 50 KB per kilometer, which is significantly smaller than the point cloud map with a size of about 600 MB per kilometer. The processed map has a relative accuracy of 9.25 cm, determined by the total station using seven sampled points.

#### 7.1.2. Localization Results

**Figure 15:**
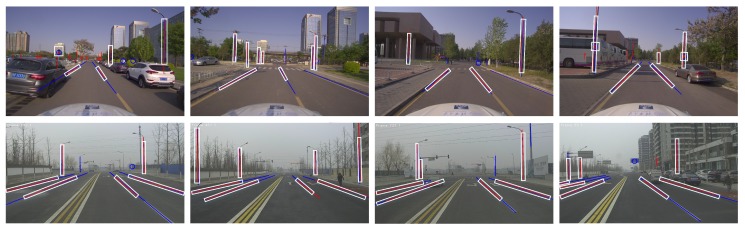
The map matching results. The top four images are from scenario 1, and the bottom 4 are from scenario 2. Recognized features are denoted in blue, and landmarks re-projected according to the result of localization are denoted in red; White boxes indicate successfully matched features.

As shown in Figure 15, the vector HD maps used in the two scenarios are re-projected to the pixel coordinate system based on the localization results before applying frame-to-frame motion fusion in order to illustrate the map matching result more intuitively. Clearly, most of the map re-projection features (red) and the image-extracted semantic features (blue) overlap each other, thereby intuitively validating the effectiveness of the map matching algorithm of MLVHM. As far as feature recognition is concerned, we can see that based on depth learning, features are not affected by occlusion. For example, roadside vehicles or trees can block some light poles, but the rest can still be recognized and provide constraints in map matching.

Features encircled by white boxes are selected by the algorithm as the association features for localization because of their high map-image correlation values; the excluded ones come from imperfect feature extractions due to image occlusion and missing map features (e.g., some lane lines). However, the selected features in each frame are more than sufficient for accurate vehicle localization, thereby validating the data association and localization algorithms of MLVHM.

**Figure 16:**
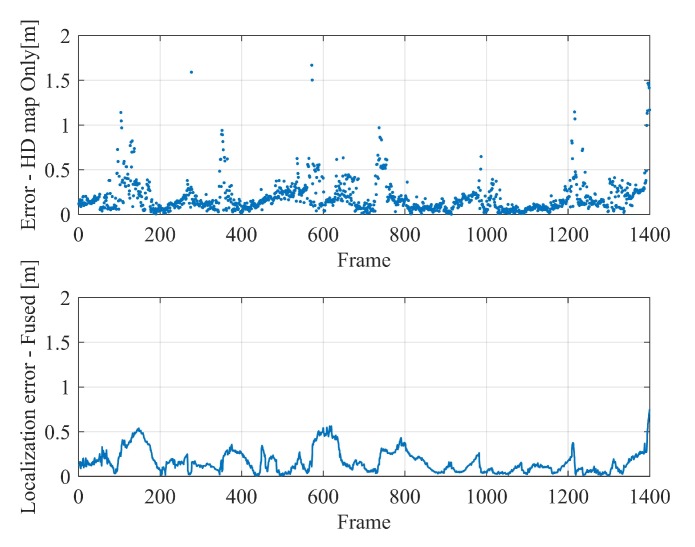
Vehicle localization results in the scenario 1—top: positioning errors of map-based localization; bottom: translation errors after fusing frame-to-frame constraints.

**Figure 17:**
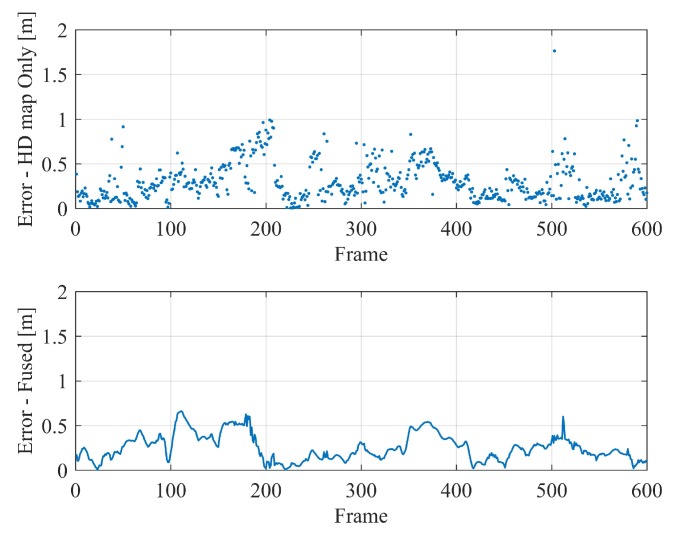
Vehicle localization results in the scenario 2—top: positioning errors of map-based localization; bottom: translation errors after fusing frame-to-frame constraints.

The resultant localization errors in the horizontal plane of the map-based localization in the two scenarios are shown in Figure 16 and Figure 17 respectively, of which the sub-figures above are the localization results based merely on HD map, while the bottom ones are those found after the frame-to-frame motion fusion. In scenario 1, the distance between landmarks is smaller, which is more conducive to map-based localization, and the positioning errors are relatively lower than that in scenario 2. In addition, in each test, when the vehicle passes through the intersection and other places with sparse features, the positioning error tends to increase. This trend, as well as the positioning accuracy, are basically consistent with our simulation experiments in the former section. This phenomenon indicates that the MLVHM localizes vehicles more effectively with denser semantic features, and implies that the distance between the vehicle and the respective features plays a critical role as well.

We give some quantitative statistical results in comparison with other methods in Table 2 and Table 3, by which we examine the localization error between the estimated camera poses and the reference trajectories. In the ORB-SLAM method, we give it the correct initial pose and scale of translation. The RMSE of localization of MLVHM in Scenario 1 and 2 are 0.21 m and 0.29 m, respectively. Detailed information including the maximum errors under 90% and 95% of the localization results, with the errors in the longitudinal and lateral direction are provided. In addition, we provide the smoothness metric proposed in [42]. A trajectory with a lower smoothness metric guarantees less outliers of localization results, and is more favorable to motion planning. The smoothness (S) we use is defined as:

(26) $$S=\frac{1}{T}\sum_{i=1}^{T}||\left(x_{i}-x_{i-1}\right)-\left(x_{i}^{\mathrm{GT}}-x_{i-1}^{\mathrm{GT}}\right)||,$$

where, $x_{i}$ is the estimated pose and $x_{i}^{\mathrm{GT}}$ is the reference pose provided by LiDAR.

Considering Table 2 and Table 3, it is not difficult to find that the localization accuracy is improved by integrating frame-to-frame motion. What’s more, the smoothness of the trajectory is greatly improved, while the largest localization error is reduced. Frame-to-frame motion fusion has lead to smoother and more stable localization results, thereby delivering high-precision localization in feature-sparse areas and localization failure-prone areas (e.g., road intersections) without the need for additional sensors.

As for ORB-SLAM2, the localization result is affected by the error accumulation in both scenarios. In the second scenario, the localization result suffers a serious scale drift caused by the unstable tracking of visual feature points. In our work, the sliding window technique solves the scale of trajectory in a piece-wise manner. By dynamically adjusting the scale, the final positioning result is improved.

In Table 4, we compare the performance of the algorithm in this paper with other map matching algorithms by their reported localization accuracy.The RMSE of our algorithm in all two scenarios is 0.24m. The map format and on-board vehicle sensors they used are also compared. From Table 4, it can be found that the MLVHM algorithm requires a lower-cost sensor configuration setup and a more lightweight HD map format, but most importantly, it delivers a better localization performance.

For intelligent vehicle planning and the autonomous control module, real-time output from the localization module is a necessity. In this experiment, we calculated the delay from the middle results as well as the final output of the localization module. We conducted test on a PC equipped with a GeForce GTX 1080 Ti graphics card paired with 12 G memory and an Intel (R) Xeon (R) E5-2620 v4 @ 2.10 GHz processor. Due to semantic segmentation in the positioning module, the average output delay of the map-based localization is 0.515 s. After fusion with the frame-to-frame constraints, the output frequency of the whole positioning algorithm is determined by the time of SLAM algorithm and the time of the sliding window optimization. The average calculated delay is 0.059 s. It can be seen that the real-time performance of the positioning algorithm is significantly improved by integrating the frame-to-frame constraints.

## 8. Conclusion and Future Works

This paper presents the MLVHM, a novel algorithm of absolute vehicle position estimation, based on low-cost monocular vision and commercialized HD maps equipped on production vehicles. The algorithm effectively achieved high localization accuracy using semantic geometry information as cues for map matching as well as frame-to-frame motion fusion. The algorithm is validated by both simulations and vehicle tests with the localization RMSE of 24 cm in typical traffic scenarios.

In future research works, road curves and other complex road patterns will be introduced into the matching error functions with their corresponding line features, thereby enabling the MLVHM to adapt to more complex traffic scenarios. Other low-cost localization sensors, such as GNSS, will also be included to help the MLVHM meet the evolving needs of intelligent vehicles.

## Figures and Tables

**Table 1:** Selected prior arts in HD map-based vehicle localization.

Map	Visual Features	Reported Accuracy	Ref
3D point map + visual features	ORB feature points	30±11 cm	[24]
3D point map	SIFT feature points	–	[5]
2D semantic vector map	Trees (stereo vision)	<30 cm	[33]
2D semantic vector map	2D lane lines and landmarks (with LiDAR)	37.1 cm	[34]
3D semantic point map	Semantic segmented pixels	<2 m	[6]

**Table 2:** Localization results in scenario 1.

Methods	Localization Error (m)			Smoothness	
	RMSE	90%	95%	Mean.	95%
Xiao et al. [35]	0.27	0.38	0.53	0.10	0.43
ORB SLAM2 [23]	0.57	0.88	1.03	0.03	0.07
**MLVHM**	**0.21**	**0.36**	**0.44**	**0.02**	**0.06**

**Table 3:** Localization results in scenario 2.

Methods	Localization Error (m)			Smoothness	
	RMSE	90%	95%	Mean.	95%
Xiao et al. [35]	0.37	0.62	0.715	0.15	0.53
ORB SLAM2 [23]	3.24	6.31	7.50	0.07	0.17
**MLVHM**	**0.29**	**0.49**	**0.53**	**0.04**	**0.12**

**Table 4:** Overall performance comparison with other methods.

Methods	On-Board Sensors	Pre-Collected Maps	Localization Error (m)
Caselitz et al. [24]	1 camera	3D Lidar point cloud map	0.30 m
Andreas Schindler et al. [34]	1 camera and Lidar and IMU	Vector map	1.00 m
Erik Stenborg et al. [6]	2 camera	3D Lidar point cloud map	0.60 m
**MLVHM**	**1 camera**	**Lightweight vector map**	**0.24 m**
